# Exploring ACC deaminase-producing bacteria for drought stress mitigation in *Brachiaria*

**DOI:** 10.3389/fpls.2025.1607697

**Published:** 2025-08-19

**Authors:** Jéssica P. Ferreira, Márcia S. Vidal, José I. Baldani

**Affiliations:** ^1^ Department of Crop Sciences, Crop Sciences Graduate Program, Federal Rural University of Rio de Janeiro (UFRRJ), Institute of Agronomy, Seropédica, Rio de Janeiro, Brazil; ^2^ Genetics and Biochemistry Laboratory, Embrapa Agrobiologia, Seropédica, Rio de Janeiro, Brazil

**Keywords:** plant growth promoting bacteria, pasture, 1-Aminocyclopropane-1-carboxylic acid, ethylene stress, inoculation, plant colonization, mCherry reporter gene, polyethylene glycol

## Abstract

Plant growth-promoting bacteria (PGPB) possessing 1-aminocyclopropane-1-carboxylate (ACC) deaminase activity have the potential to enhance plant growth and development, particularly under adverse environmental conditions. This study aimed to identify bacterial strains with ACC deaminase activity able of mitigating the effects of water deficit stress and promoting the growth of *Brachiaria* genotypes. Bacterial strains isolated from *Brachiaria* genotypes were screened *in vitro* for ACC deaminase activity, and the presence of the *acdS* gene was confirmed via polymerase chain reaction (PCR) analysis. The bacterial isolates were screened for *in vitro* tolerance to water deficit stress, using 10% polyethylene glycol 8000 (PEG 8000) in association with *B. ruziziensis* and the effects of bacterial inoculation were assessed based on plant height and fresh biomass accumulation. Additionally, the association between endophytic bacterial strains and *Brachiaria* genotypes was evaluated using confocal laser microscope. The results showed that among the 213 strains tested, 32 demonstrate the ability to degrade ACC into α-ketobutyrate. ACC deaminase activity was detected in 17 strains, with values ranging from 1.98 to 102.52 μmol α-ketobutyrate mg^-1^ protein h^-1^. The presence of the *acd*S gene was confirmed in nine strains. The strains NRB142 (*Paraburkholderia silvatlantica*), NRB223 (*Azospirillum melinis*), and BR11790 (*Herbaspirillum frisingense* GSF30^T^) exhibited the most significant promotion of plant development in *B*. *ruziziensis* under water deficit stress mediated by 10% PEG 8000. Confocal microscopy analysis revealed the rhizospheric and inner root colonization of *B*. *ruziziensis* and *B*. *brizantha* cv. Paiaguás by the NRB142 mCherry-labeled strain. This study showed no predominance of a specific group of bacterial strains in terms of ACC deaminase activity. However, a subset of strains demonstrated the ability to colonize *Brachiaria* plants and mitigate the negative effects of water deficit stress. This study highlights the potential of ACC deaminase-producing bacteria in alleviating water deficit stress in *Brachiaria* plants supporting their use as a promising strategy for improving plant resilience under drought conditions.

## 1 Introduction

The Brazilian cattle herd is estimated to be the second largest in the world, with approximately 186.8 million head in 2025 (USDA, 2025). The herd is predominantly fed through free grazing on pastures, which remains the most economical and practical method of providing nutrition for livestock (Jank et al., 2014; Duarte et al., 2020). Among the pasture areas in Brazil, it is estimated that approximately 85% are occupied by plants of the *Brachiaria* genus (Jank et al., 2014).

Abiotic stresses are recognized as a primary influence impacting agricultural production globally (Shahid et al., 2023). Among abiotic stresses, water stress is the one that most frequently affects pasture productivity (Moore et al., 2020). Water is vital for plant development (Shao et al., 2008), and its scarcity can disrupt growth cycles, leading to yield reductions exceeding 50% (Boyer, 1982; Lisar et al., 2012). Water stress impacts various levels of plant organization (Yordanov et al., 2000), altering water potential, turgor pressure, nutrient transport, and gas exchange. Increased abscisic acid levels cause stomatal closure, inhibiting photosynthesis. This leads to the accumulation of Reactive Oxygen Species (ROS), causing cellular damage such as DNA lesions, protein synthesis inhibition, pigment oxidation, and membrane deterioration. The decline in chlorophyll content, often linked to oxidative stress, further hampers photosynthetic efficiency (Anjum et al., 2011; Taiz and Zeiger, 2017; Vurukonda et al., 2016). Additionally, water deficit compromises cell wall and membrane integrity, leading to cell death (Ali et al., 2025). These effects reduce plant growth, accelerate senescence, decrease dry matter production, and heighten susceptibility to diseases and pests, ultimately diminishing crop quality and yield (Li et al., 2009; Seleiman et al., 2021).

The application of microbial inoculants containing plant growth-promoting bacteria (PGPB) presents a cost-effective and environmentally sustainable solution to mitigate water deficit stress in crops (Poudel et al., 2021; Armanhi et al., 2021). PGPB enhance plant growth while offering protection against diseases and abiotic stresses, including drought, salinity, and nutrient imbalances (Dimkpa et al., 2009; Grover et al., 2011; Glick, 2012, 2015). In this context, the use of microorganisms is important due to their low cost and eco-friendly nature make them an attractive option for sustainable agriculture. Additionally, PGPB contribute to overall plant health, increase productivity and quality, and help maintain soil integrity (Zhang et al., 2019). Harnessing their potential can lead to more resilient crop systems, reducing the adverse impacts of water deficit stress while supporting sustainable farming practices.

Plant growth-promoting bacteria enhance plant tolerance to water stress through several key mechanisms. They improve nutrient availability via biological nitrogen fixation, phosphate solubilization, and siderophore production which aids iron uptake (Ali and Khan, 2021; Sati et al., 2023). Additionally, PGPB produce exopolysaccharides, which enhance soil structure, water retention, and root stability (Ali and Khan, 2021; Sati et al., 2023). They also regulate osmotic balance and activate antioxidant defenses, mitigating reactive oxygen species (ROS) damage and protecting biomolecules from oxidative stress (Grover et al., 2011; Sati et al., 2023). Furthermore, PGPB facilitates the synthesis of heat shock proteins (HSPs), dehydrins, and volatile organic compounds (VOCs), which assist in drought tolerance and adaptation (Kaushal and Wani, 2016). Their influence on phytohormone production, including auxins, gibberellins, cytokinins, abscisic acid, and ethylene, helps regulate plant growth under water deficit conditions (Kaushal and Wani, 2016).

Ethylene stress, triggered by abiotic and biotic factors, adversely affects plant growth, causing senescence, abscission, chlorophyll loss, and developmental inhibition (Pei et al., 2017; Glick et al., 2007a; Depaepe and van der Straeten, 2016; Kaushal and Wani, 2016). Plant growth-promoting bacteria (PGPB) expressing 1-aminocyclopropane-1-carboxylate (ACC) deaminase counteract excessive ethylene by cleaving ACC (the immediate precursor of ethylene) into ammonia and α-ketobutyrate (Glick et al., 1998). The *acd*S gene encoding ACC deaminase has been identified in diverse organisms within the Eukarya, Bacteria, and Archaea domains. However, it predominantly occurs in various bacterial species and some fungi (Singh et al., 2015; Soni et al., 2018). The products of enzymatic cleavage, ammonia and α-ketobutyrate, serve as carbon and nitrogen sources for bacterial metabolism (Glick and Nascimento, 2021). Additionally, α-ketobutyrate influences the transcriptional regulation of *acd*S, a gene involved in ethylene modulation, through its role in leucine biosynthesis (Soni et al., 2018). The *acd*S gene is regulated by AcdR (Lrp), a leucine-responsive protein, and co-regulated by AcdB, which forms a complex with ACC and Lrp, enabling *acd*S transcription (Glick et al., 2007a; Li and Glick, 2001; Cheng et al., 2008). Upon ACC deaminase synthesis, ACC is cleaved, generating ammonia and α-ketobutyrate, which is then used for leucine biosynthesis. As leucine accumulates, it binds to Lrp octamer, causing its dissociation into inactive dimers, ultimately blocking *acd*S transcription. This regulatory mechanism ensures that ACC deaminase is produced only when required (Cheng et al., 2008; Duan et al., 2013; Glick et al., 2007b; Grichko and Glick, 2000; Li and Glick, 2001). Therefore, this enzymatic action may help mitigate the stress effects of ethylene on plants colonized by these bacteria, enhancing growth by reducing its inhibitory influence. The fate of these byproducts seems to be important in microbial interactions with plants, supporting healthier development in challenging environments.

The increasing frequency of droughts due to climate change has intensified research on plant interactions with ACC deaminase-producing bacteria. Chandra et al. (2019, 2020) found that *Variovorax paradoxus* and a consortium of *Ochrobactrum anthropi*, *Pseudomonas palleroniana*, and *Pseudomonas fluorescens* improved millet and wheat growth under 35% water stress, increasing leaf nutrient concentrations and antioxidant properties. Similarly, Tahir et al. (2019) reported that corn inoculated with BN-5 and MD-23 strains showed enhanced grain yield, relative water content, and chlorophyll levels under 50% field capacity. Likewise, Tiwari et al. (2018) analyzed drought and saline stress mitigation in *Panicum maximum* inoculated with ACC deaminase-producing rhizobacteria, and observed improving membrane stability, solute accumulation, and photosynthetic pigments while Ojuederie and Babalola (2023) found that *Pseudomonas* sp. MRBP4, MRBP13, and *Bacillus* sp. MRBP10 strains enhanced maize drought tolerance by improving water retention and biomass production. Similarly, Jasso-Arreola et al. (2025) demonstrated that *Pantoea* sp. RCa62, isolated from *Coffea arabica*, increased leaf area, root development, and relative water content while reducing proline accumulation. Other studies have confirmed similar benefits in various crops, including tomato (Muñoz-Carvajal et al., 2024), soybean (Dubey et al., 2024), black gram (Chandwani and Amaresan, 2024), watermelon (Yavuz et al., 2023), and cluster bean (Jain and Saraf, 2023). These findings reinforce the potential of ACC deaminase-producing bacteria as a biological strategy for mitigating drought stress and promoting sustainable agriculture.

Therefore, this study aimed to identify diazotrophic bacteria with ACC deaminase activity that can alleviate water deficit stress and promote the growth of *Brachiaria* genotypes under stress conditions induced by polyethylene glycol 8000 (PEG 8000). Additionally, it assessed the ability of the selected bacterium to colonize and establish in *Brachiaria ruziziensis* plants grown *in vitro* under PEG 8000 treatment.

## 2 Materials and methods

### 2.1 Qualitative screening for bacterial strains with ACC deaminase activity

The screening for ACC deaminase activity in the strains was conducted following the approach described by Glick et al. (1995), with minor modifications. The study involved the analysis of 213 diazotrophic strains isolated from various *Brachiaria* genotypes as part of the Embrapa project (number 02.13.08.004.00.00). The isolation, the taxonomic and partial functional characterization of these 213 strains were previously reported by Ribeiro et al. (2020).

The strains were cultivated in 5 mL of DYGS medium (Rodrigues Neto et al., 1986) and incubated at 30°C, 180 rpm, for 24 or 48 h, depending on the bacterial growth rate. Following incubation, 100 µL of the culture was transferred to new tubes containing 5 mL of LGI or NFb medium (Baldani et al., 2014) supplemented with 1 g L⁻¹ of (NH_4_)_2_SO_4_ as a nitrogen source and incubated under the same conditions. Afterward, 100 µL of the second-round culture was transferred to fresh tubes containing 5 mL of LGI or NFB medium, but without the nitrogen source. The culture medium was then supplemented with 3 mmol L⁻¹ ACC and incubated under the previously described conditions.

Petri dishes containing Noble Agar (low nitrogen content) were supplemented with 3 mmol L⁻¹ ACC from a filtered sterilized stock solution (0.5 mol L⁻¹), which was evenly spread over the surface of the culture medium. Cultures were inoculated using a sterile cotton swab and incubated at 30°C for 48 or 60 h, depending on growth conditions. For the negative control, cultures were plated on LGI or NFb media without the addition of inorganic nitrogen or ACC substrate. A diazotrophic *Herbaspirillum frisingense* strain GSF30^T^, recognized for its ACC deaminase activity, was used as a positive control (Rothballer et al., 2008).

### 2.2 Quantification of ACC deaminase activity produced by the pre-selected strains

The activity of ACC deaminase was assessed using the method described by Penrose and Glick (2003), which quantifies the α-ketobutyrate produced through the cleaved by the enzyme ACC deaminase. The quantification process involved measuring the absorbance of bacterial sample at 540 nm and comparing the results to a standard α-ketobutyrate curve ranging from 10 to 1000 µmol. To determine the specific activity of the cultures, protein concentration was measured using the Bradford method (Bradford, 1976).

The pre-selected strains (qualitative assays) were cultured overnight in 5 mL of DYGS medium at 30°C, 180 rpm, for 24 h. After incubation, the cells were harvested by centrifugation at 5,000 xg for 10 min at 4°C, followed by washing with NFb or LGI medium (without a nitrogen source). The bacterial pellet was then resuspended in 5 mL of NFb or LGI medium supplemented with 3 mmol L⁻¹ ACC as the sole nitrogen source. The culture was incubated for 24 h with shaking at 180 rpm at 30°C. Subsequently, the bacterial cells were harvested again by centrifugation at 5,000 xg, 4°C, for 10 min. The cells were washed twice with 5 mL of 0.1 mol L⁻¹ Tris-HCl buffer (pH 7.6). Finally, the cell suspension was transferred to a microcentrifuge tube and centrifuged at 10,000 g for 1 min.

All the supernatant was carefully removed, and the cell pellet was utilized for the enzymatic assay. The pellets were resuspended in 400 μL of 0.1 mol L⁻¹ Tris-HCl buffer (pH 8.0), followed by the addition of 20 μL of toluene and vortexing for 30 s. Subsequently, 50 μL of the toluene-treated cells were incubated with 5 μL of 0.5 M ACC at 30°C for 30 min. After incubation, 500 µL of 0.56 M HCl was added, and the mixture was vortexed and centrifuged at 10,000 xg for 5 min at room temperature. The resulting supernatant (500 μL) was vortexed with 400 μL of 0.56 M HCl and 150 μL of 2,4-dinitrophenylhydrazine reagent (0.2% 2,4-dinitrophenylhydrazine in 2 M HCl). The mixture was incubated at 30°C for 30 min, followed by the addition of 1 mL of 2 M NaOH and thorough mixing. Absorbance at 540 nm was then measured using a spectrophotometer. The cell suspension without ACC served as the negative control. Specific activity of the cultures was determined by protein quantification following the Bradford method (Bradford, 1976). ACC deaminase activity was expressed as μmol of α-ketobutyrate per mg of protein per hour.

### 2.3 Detection of the *acd*S sequence in the bacterial strains

The positive strains in the qualitative screening and those that showed results in the quantification of ACC deaminase enzyme activity were used in the detection of the *acd*S sequence. Genomic DNA was extracted using the commercial Wizard^®^ Genomic DNA Purification Kit (Promega, Madison, USA) following the manufacturer’s instructions. The concentration of genomic DNA was evaluated using a Nanodrop^®^ 3300 spectrophotometer (Thermo Fisher Scientific Inc., Waltham, USA). PCR reactions were performed using two pairs of primers described by Li et al. (2015): acdSf3 (5′ – ATCGGCGGCATCCAGWSNAAYCANAC – 3′), acdSr3 (5′ – GTGCATCGACTTGCCCTCRTANACNGGRT – 3′), and acdSr4 (5′ – GGCACGCCGCCCARRTGNRCRTA – 3′). Each amplification reaction was conducted in a final volume of 25 μL, consisting of 20 ng µL⁻¹ genomic DNA, 1× Taq DNA polymerase buffer (1 mM Tris-HCl, pH 9.0, and 5 mM KCl), 0.5 mM of each dNTP, 3 mM MgCl_2_, 0.4 μM of each primer, and 0.1 U µL⁻¹ Taq DNA polymerase (Promega, Madison, USA). Negative control samples were prepared by replacing bacterial DNA with ultrapure water. Amplification reactions were carried out in a SureCycler 8800 thermocycler (Agilent Technologies, Santa Clara, USA) programmed for initial denaturation at 94°C for 4 min; 35 cycles at 94°C for 45 s, 53°C for 45 s, and 72°C for 1 min; followed by a final extension at 72°C for 10 min. After amplification, 2 μL of PCR product was analyzed through electrophoresis on a 1.5% (w/v) agarose gel at 90 volts (~5 V/cm) for 1 h and 30 min in 1× TAE buffer (40 mM Tris-acetate, pH 8.0, and 1 mM EDTA, pH 8.0). The gel was stained with ethidium bromide solution (0.5 μg mL⁻¹) and visualized under ultraviolet light using a KODAK^®^ Gel Logic Cabinet 100 photoceller (Eastman Kodak Company, Rochester, USA).

### 2.4 *In vitro* response of *B. ruziziensis* to inoculation with diazotrophic ACC deaminase-producing strains under stress conditions

A preliminary experiment was conducted to determine the optimal PEG 8000 concentration for *in vitro* studies. The application of 20% PEG 8000 was highly detrimental, leading to the death of nearly all plants (data not shown). Based on these results, a subsequent experiment was performed using a reduced concentration of 10% PEG 8000.

The gnotobiotic inoculation experiment was conducted using disinfested seeds of *Brachiaria ruziziensis*, a genotype with low tolerance to water deficit stress. The seeds were peeled and sterilized by washing in 70% (v/v) ethanol for 3 min, followed by immersion in sodium hypochlorite (4–6% v/v free chlorine) with agitation for 10 min. After that, the seeds were rinsed three times with sterile distilled water and placed in Petri dishes containing an agar/water medium (0.5% agar supplemented with 500 mg L⁻¹ of yeast extract). The plates were initially incubated in the dark at 30°C for 24 h. Subsequently, they were transferred to a BOD incubator (model LB41, LABTEC, Londrina, Paraná, Brazil) and maintained at 30°C with a 12-h photoperiod for 4 days to ensure complete germination.

Meanwhile, the bacterial strains were inoculated in 50 mL of liquid DYGS medium and incubated under agitation at 180 rpm and 30°C, for 24 or 48 h, depending on the bacterial growth rate. Bacterial growth was quantified using the micro drop technique (Romeiro, 2007). The inoculum concentrations obtained were 10^4^ CFU mL⁻¹ for strain BR11790 and 10^5^ CFU mL⁻¹ for the other target diazotrophic bacteria. Non-contaminated seedlings were carefully removed from the agar/water medium and immersed for 1 h in the bacterial culture suspension of their respective strains: NRB032 (*Stenotrophomonas maltophilia*), NRB039 (*Nitrospirillum amazonense*), NRB058 (*Pseudomonas cremoricolorata*), NRB096 (*Bacillus safensis*), NRB123 (*N*. *amazonense*), NRB124 (*Paraburkholderia silvatlantica*), NRB127 (*Herbaspirillum seropedicae*), NRB138 (*Gluconacetobacter diazotrophicus*), NRB142 (*P*. *silvatlantica*), NRB223 (*Azospirillum melinis*) and BR11790 (*H. frisingense* GSF30^T^). These strains were employed as they showed positive results in the qualitative screening and demonstrated activity in the quantification of the ACC deaminase enzyme. A *Herbaspirillum frisingense* strain GSF30^T^ was used as a positive control. The seedlings assigned to the control treatment were immersed in flasks containing the same volume of DYGS liquid medium for the same duration. Afterward, the inoculated seedlings were transferred to glass tubes containing 25 mL of MS medium (Murashige and Skoog, 1962) supplemented with 30 g L⁻¹ of sucrose, with the pH adjusted to 5.8. The medium was prepared both with and without 10% PEG 8000. The plants were then placed in a growth room and maintained for 30 days under a photoperiod of 16 h of light and 8 h of darkness, at a constant temperature of 25°C.

One experiment was conducted in a completely randomized design with four replications. The factors included stress induction mediated by PEG 8000 (present or absent), two seed treatments (inoculation with 11 diazotrophic strains exhibiting ACC deaminase activity and a control), and one *Brachiaria* genotype (*B*. *ruziziensis*). Each experimental unit consisted of a 100 mL glass tube containing 25 mL of MS medium. Analyses were performed 30 days post-inoculation by measuring plant height and the fresh biomass accumulation of leaves and roots. To compare treatment means, the Scott-Knott test was applied at a significant level of 0.05. All statistical analyses were conducted using the software ‘Sisvar’ version 5.3 (Ferreira, 2011).

### 2.5 Assessment of bacterial colonization in *B. brizantha* cv. Paiaguás and *B. ruziziensis*

To assess the *Brachiaria* plant colonization, a red-fluorescent derivative of NRB142 (*P*. *silvatlantica*) was constructed via transformation with plasmid pLMB426 applying the electroporation method. The transformed strain, designated NRB142 (mCherry), was cultivated in liquid or solid DYGS medium supplemented with gentamycin (80 μg mL⁻¹). Plants of *B*. *brizantha* cv. Paiaguás and *B*. *ruziziensis* with 5 days after germination were inoculated with NRB142 (mCherry). This strain was selected because it exhibited the highest performance in ACC deaminase activity quantification and showed beneficial effects in the *in vitro* test with PEG 8000. The gnotobiotic inoculation experiment utilized disinfected seeds of *B*. *brizantha* cv. Paiaguás and *B*. *ruziziensis*, as described in the previous section.

After germination, microorganism-free plants were removed from agar/water medium plates and transferred to glass tubes containing 25 mL of MS medium (Murashige and Skoog, 1962) supplemented with 30 g L⁻¹ sucrose and adjusted to pH 5.8 for rooting. Plants were maintained in MS medium for 30 days to promote root formation before being transferred to flasks containing 25 mL of Hoagland’s solution. Prior to transfer, inoculation with NRB142 (mCherry) was conducted in tubes designed for the inoculated treatment, using bacterial suspensions prepared at a concentration of 10^5^ CFU mL⁻¹. Bacterial growth was quantified using a Neubauer chamber.

Control treatment tubes were inoculated with PBS buffer in volumes equal to the bacterial solution. The experiment followed a completely randomized design with four replications, considering two experimental factors: inoculation with or without NRB142 (mCherry) and two *Brachiaria* cultivars (*B*. *brizantha* cv. Paiaguás and *B*. *ruziziensis*). Each experimental unit consisted of a 100 mL glass tube containing 25 mL of MS medium (Murashige and Skoog, 1962).

Plants were maintained in a growth chamber for 30 days under a controlled photoperiod of 16 h of light and 8 h of darkness at 25°C. Harvests were performed at 3, 7, and 14 days after inoculation (dai). Endophytic and rhizospheric bacterial populations were quantified using the micro-drop technique (Romeiro, 2007). Confocal microscopy images were obtained using the LSM 700 microscope, AxioObserver (Carl Zeiss, Jena, Germany), and processed with Zen 2.3 software (Carl Zeiss, Jena, Germany).

## 3 Results

### 3.1 Screening for bacterial strains with ACC deaminase activity

The methodology adapted from Glick et al. (1995) was initially used to assess the presence of ACC deaminase activity in bacterial strains. Some strains exhibited growth in LGI or NFb agar plates with ACC as the sole nitrogen source, indicating positive ACC deaminase activity. The results indicated that bacterial growth relied on ACC as its sole nitrogen source, consistent with the methodology described by Glick et al. (1995). Screening of the 213 bacterial strains isolated from *Brachiaria* genotypes revealed that approximately 15% possessed ACC deaminase activity. Among these, 25% were isolated from rhizospheric soil, 25% from disinfected roots, and 50% from non-disinfected roots (**Table 1**).

**Table 1:** Quantification of ACC deaminase activity produced by different strains originally isolated from *Brachiaria* genotypes.

Strain	Taxonomic identification*	Origin of the strain	Tissue	ACC deaminase activity (μmol α-ketobutyrate mg^-1^ protein h^-1^)	*acdS* gene
NRB142	*Paraburkholderia silvatlantica*	*B. brizantha* (Marandu)	SR	102,52	+
NRB138	*Gluconacetobacter diazotrophicus*	*B. decumbens* (D24/27)	SR	89,49	+
NRB053	*Nitrospirillum amazonense*	*B. decumbens*	RD	77,49	–
NRB123	*Nitrospirillum amazonense*	*B. decumbens* (Basilisk)	RD	63,57	–
NRB039	*Nitrospirillum amazonense*	*B. decumbens*	RND	59,41	–
NRB058	*Pseudomonas cremoricolorata*	*B. decumbens* (D24/27)	RD	49,30	+
NRB124	*Paraburkholderia silvatlantica*	*B. brizantha* (B140)	RND	47,53	–
NRB121	*Nitrospirillum amazonense*	*B. decumbens* (Basilisk)	SR	45,63	–
NRB127	*Herbaspirillum seropedicae*	*B. decumbens* (D24/27)	RND	44,83	+
NRB059	*Bacillus aerius*	*B. decumbens*	SR	30,74	–
NRB087	*Azospirillum oryzae*	Hybrid Mulato (*B*. *ruziziensis* x *B*. *brizantha* cv. Marandu)	RD	16,44	+
NRB223	*Azospirillum melinis*	*B. decumbens*	SR	8,90	+
NRB135	*Nitrospirillum amazonense*	*B. decumbens*	RD	6,20	–
NRB096	*Bacillus safensis*	*B. humidicola* (Tupi)	RND	4,73	+
NRB032	*Stenotrophomonas maltophilia*	*B. decumbens* (D24/25)	RND	3,14	+
NRB093	*Pseudomonas geniculata*	*B. humidicola* (Tupi)	RND	2,55	–
NRB086	*Azospirillum lipoferum*	*B.* spp. (H331 – Ipyporam)	SR	1,98	+
NRB024	*Azospirillum lipoferum*	*B. decumbens* (D24/27)	RND	n.d.	–
NRB030	*Bacillus aerius*	*B. decumbens*	SR	n.d.	–
NRB082	*Azospirillum formosense*	*B. decumbens*	RND	n.d.	–
NRB102	*Stenotrophomonas maltophilia*	*B. decumbens* (D24/27)	RND	n.d.	–
NRB111	*Paraburkholderia silvatlantica*	*B*. spp. (H331 – Ipyporam)	RND	n.d.	–
NRB128	*Nitrospirillum amazonense*	*B. decumbens* (D24/27)	RD	n.d.	–
NRB153	*Nitrospirillum amazonense*	*B. brizantha* (Xaraés)	RD	n.d.	–
NRB157	*Nitrospirillum amazonense*	*B. brizantha* (Xaraés)	RND	n.d.	–
NRB190	*Burkholderia tropica*	*B. decumbens* (D24/2)	RND	n.d.	–
NRB208	*Azospirillum brasilense*	*B. decumbens*	RND	n.d.	–
NRB211	*Pseudomonas kuykendallii*	*B. brizantha* (Xaraés)	SR	n.d.	–
NRB214	*Flavobacterium anhuiense*	*B. decumbens* (D24/27)	RND	n.d.	–
NRB218	*Stenotrophomonas maltophilia*	*B. brizantha* (Xaraés)	RND	n.d.	–
NRB225	*Stenotrophomonas maltophilia*	*B. brizantha* (Piatã)	RND	n.d.	–
NRB227	*Bacillus subtilis*	*B. brizantha* (Paiaguás)	RD	n.d.	–
GSF30**	*Herbaspirillum frisingense*	*Miscanthus sacchariflorus*	F	9,28	+

NDR, non-disinfested root; DR, Disinfested root; RS, Rhizospheric soil; L, Leaf; n.d., not detected.

*Ribeiro et al. (2020)

**Rothballer et al. (2008)

### 3.2 Quantification of ACC deaminase activity

The activity of the enzyme ACC deaminase was determined by quantifying the α-ketobutyrate produced during the deamination of ACC by the enzyme. In this work, 32 strains that showed growth capacity in ACC culture medium as the sole nitrogen source were selected to quantify the activity of the ACC deaminase enzyme. The results indicate that 17 out of the 32 strains exhibited ACC deaminase activity *in vitro* (**Table 1**), while the remaining 15 strains tested negative for ACC deaminase activity. These findings suggest that growth in a medium with ACC as the sole nitrogen source is not sufficient to confirm that a bacterial strain possesses ACC deaminase activity. Therefore, it is essential to quantify ACC enzyme activity to verify its presence.

As expected, the positive control, *H*. *frisingense* GSF30^T^, exhibited ACC deaminase activity of 9.28 μmol α-ketobutyrate mg^-1^ protein h^-1^ in the present assay. Another species of the same genus, *H*. *seropedicae* (NRB127), showed higher activity than *H*. *frisingense* GSF30^T^, with a value of 44.83 μmol α-ketobutyrate mg^-1^ protein h^-1^. The species *P*. *silvatlantica* (NRB142) and *G*. *diazotrophicus* (NRB138) showed the highest ACC deaminase activities *in vitro*, with values of 102.52 and 89.49 μmol α-ketobutyrate mg^-1^ protein h^-1^, respectively. Among the results obtained, a group of bacteria presented intermediate ACC deaminase activity values, ranging from 49.0 to 16.0 μmol α-ketobutyrate mg^-1^ protein h^-1^. For instance, strain NRB058 (*P*. *cremoricolorata*) exhibited an activity of 49.30 μmol α-ketobutyrate mg^-1^ protein h^-1^ whereas the strain NRB087 (*A*. *oryzae*) showed a lower value of 16.44 μmol α-ketobutyrate mg^-1^ protein h^-1^. Regarding the lowest enzymatic activity values, results ranged from 8.90 to 1.98 μmol α-ketobutyrate mg⁻¹ protein h⁻¹. The lowest observed enzymatic activity was recorded for strain NRB086, with a value of 1.98 μmol α-ketobutyrate mg⁻¹ protein h⁻¹.

### 3.3 Detection of the *acd*S sequence in the genome of these bacterial strains

**Figure 1:**
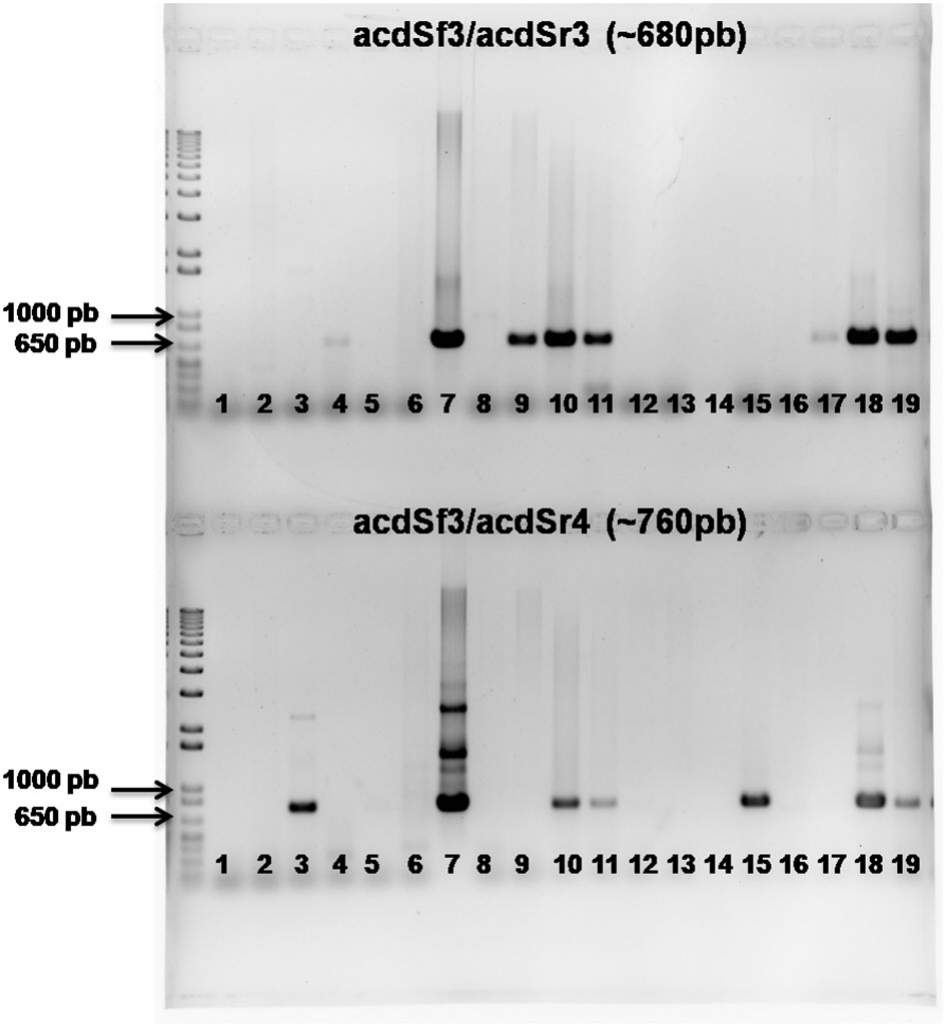
Amplification of the *acdS* gene from the chromosomal DNA of bacterial strains with ACC deaminase activity. M: Marker 1 kb Plus DNA ladder; Line 1: Water for PCR; Line 2: Negative control; Line 3: BR11790; Line 4: NRB032; Line 5: NRB039; Line 6: NRB053; Line 7: NRB058; Line 8: NRB059; Line 9: NRB086; Line 10: NRB087; Line 11: NRB096; Line 12: NRB121; Line 13: NRB123; Line 14: NRB124; Line 15: NRB127; Line 16: NRB135; Line 17: NRB138; Line 18: NRB142; Line 19: NRB223. The upper part of the gel shows the amplified product using the acdSf3/acdSr3 primers, while the bottom gel shows the combination of primers acdSf3/acdSr4.

The predicted amplified PCR products (~ 680 bp with acdSf3/acdSr3 or ~ 760 bp with acdSf3/acdSr4) were successfully obtained for 9 bacterial strains exhibiting ACC deaminase: NRB032 (*S. maltophilia*), NRB058 (*P. cremoricolorata*), NRB086 (*A. lipoferum*), NRB087 (*A. oryzae*), NRB096 (*B. safensis*), NRB127 (*H. seropedicae*), NRB138 (*G. diazotrophicus*), NRB142 (*P. silvatlantica*) and NRB223 (*A. melinis*). Additionally, amplification was observed for the positive control *H. frisigense* GSF30^T^ (BR11790). In contrast, no amplification of the *acd*S gene was detected in the negative control (blank sample). An agarose gel electrophoresis illustrating the respective amplified product is shown in **Figure 1**.

### 3.4 ACC deaminase-producing bacteria mitigating water deficit stress in *Brachiaria ruziziensis* grown *in vitro*

The growth of *B*. *ruziziensis* in the presence of 10% PEG8000 was considerably decreased compared to control treatment (no PEG8000), which exhibited higher values for the analyzed variables (**Table 2**). There was a statistically significant difference (*p <0.05*) was observed in plant size between the control treatment and those subjected to water deficit stress. However, under water deficit stress, inoculation with strains NRB223 (*A*. *melinis*), BR11790 (*H*. *frisigense* GSF30^T^), NRB142 (*P*. *silvatlantica*), NRB032 (*S*. *maltophilia*) and NRB127 (*H*. *seropedicae*) lead to an increase in plant size, statistically differing from the other strains and the uninoculated plants. Among these plants inoculated with strain NRB223 (*A*. *melinis*) exhibited the greatest height (45.00 cm), while the shortest height (9.00 cm) was recorded in the uninoculated plants.

**Table 2:** Effect of ACC deaminase-producing bacteria inoculated in *B. ruziziensis* plants subjected to stress induced by polyethylene glycol 8000 - *in vitro* assay.

Strains	Length (cm)		Fresh Weight (g)	
	Control	Stress	Control	Stress
Uninoculated	55.60 Aa	9.00 Db	3.50 Ca	0.03 Bb
NRB032	55.70 Aa	32.80 Bb	4.70 Ba	0.30 Bb
NRB039	53.60 Aa	12.20 Db	6.20 Aa	0.08 Bb
NRB058	51.30 Aa	10.80 Db	2.60 Da	0.04 Bb
NRB096	52.40 Aa	15.00 Db	4.60 Ba	0.18 Bb
NRB123	42.60 Ba	9.90 Db	2.00 Ea	0.04 Bb
NRB124	56.30 Aa	11.20 Db	2.90 Ca	0.83 Ab
NRB127	46.70 Ba	23.20 Cb	1.70 Ea	0.14 Bb
NRB138	55.30 Aa	12.80 Db	5.30 Ba	0.06 Bb
NRB142	52.70 Aa	36.40 Bb	3.90 Ca	1.40 Ab
NRB223	55.00 Aa	45.00 Ab	3.70 Ca	1.50 Ab
BR11790	59.40 Aa	43.00 Ab	3.40 Ca	1.30 Ab
VC (%)	10.66		21.11	

Different uppercase letters indicate a statistical difference between strains. Different lowercase letters indicate differences between stress levels. Scott-Knott (*p <0.05*).

In the absence of PEG8000, no statistically significant differences were observed among the inoculated treatments and the control, except for strains NRB123 (*N. amazonense*) and NRB127 (*H*. *seropedicae*), which showed smaller plant sizes (**Table 2**). Plants inoculated with strains NRB223 (*A*. *melinis*), NRB124 (*P*. *silvatlantica*), NRB032 (*S*. *maltophilia*) showed a higher increase in plant size, reaching up to 59.40 cm. In contrast, plants inoculated with strains NRB127 (*H*. *seropedicae*) and NRB123 (*N*. *amazonense*) displayed comparatively smaller sizes, measuring 46.70 and 42.60 cm, respectively.

A statistically significant difference (*p <0.05*) was observed in fresh biomass accumulation both under control (without PEG8000) and in treatments subjected to water deficit stress (with PEG 8000). Under control conditions, plants inoculated with strain NRB039 (*N*. *amazonense*) showed the highest fresh biomass accumulation (6.20 g), differing statistically from the other inoculated strains. In contrast, plants inoculated with the strain NRB127 (*H*. *seropedicae*) accumulated the lower fresh biomass accumulation (1.70 g). Under water deficit stress, plants inoculated with strain NRB223 (*A*. *melinis*) accumulated the highest fresh biomass (1.50 g), followed by those inoculated with strains NRB142 (*P*. *silvatlantica*), BR11790 (*H*. *frisigense* GSF30^T^), and NRB124 (*P*. *silvatlantica*). These inoculated plants differed statistically from plants inoculated with other strains and the uninoculated plants, which accumulated only 0.03g of biomass.

### 3.5 Colonization of *Brachiaria* genotypes by ACC-producing strain NRB142

The ability of strain NRB142 (*P*. *silvatlantica*) to colonize seedlings of *B*. *brizantha* cv. Paiaguás and *B*. *ruziziensis* was investigated under *in vitro* conditions. The NRB142 strain was selected for this experiment as it demonstrated the highest activity in the quantification of ACC deaminase enzyme activity. Additionally, this strain also contributed to plant tolerance against water deficit stress, induced by PEG *in vitro*. The strain was successfully labeled with the plasmid harboring the *mCherry* gene, which remained stable throughout the colonization study, as confirmed by bacterial counting and microscopy analysis conducted at 3, 7, and 14 days after inoculation (dai).

Bacterial counts showed that non-disinfected roots (NDR) presented a higher bacterial colonizing the root system compared to disinfested roots (DR) in both *Brachiaria* genotypes (**Table 3**). Differences in bacterial colonization were observed between both genotypes. At 3 dai, the Paiaguás genotype showed a greater bacterial population in both NDR or DR root system. By day 7, this pattern persisted for NDR, while that disinfected roots (DR) of the Ruziziensis genotype showed a higher bacterial population (4 × 10^4^ cells g^-1^ fresh tissues). By 14 dai, a highest colony forming unit (CFU) g^-1^ fresh tissue was observed for NDR of the Ruziziensis genotype, whereas DR of the Paiaguás genotype exhibited the highest bacterial count. Despite these differences, results confirmed that strain NRB142 (*P*. *silvatlantica*) effectively colonizes both *Brachiaria* genotypes in numbers relatively high, including the root interior. Despite these differences, results confirm that strain NRB142 effectively colonizes both *Brachiaria* genotypes, including root interior colonization.

**Table 3:** Bacterial counting in roots of *Brachiaria brizantha* cv. Paiaguás and *B. ruziziensis* inoculated with NRB142(mCherry).

Treatment applied to the root	*Brachiaria brizantha* cv. Paiaguás						*Brachiaria ruziziensis*					
	3 dai		7 dai		14 dai		3 dai		7 dai		14 dai	
	× 10^6^ cells g^-1^ fresh tissues						× 10^6^ cells g^-1^ fresh tissues					
	C	I	C	I	C	I	C	I	C	I	C	I
NDR	n.d.	6.75	n.d.	4.69	n.d.	3.28	n.d.	2.12	n.d.	2.78	n.d.	4.08
DR	n.d.	0.07	n.d.	0.008	n.d.	0.06	n.d.	0.03	n.d.	0.04	n.d.	0.002

C, control; I, inoculated; NDR, non-disinfested root; DR, disinfested roots; n.d., not detected.

Values are expressed as CFU g^-1^ obtained at 3, 7 and 14 days after inoculation (dai).

**Figure 2:**
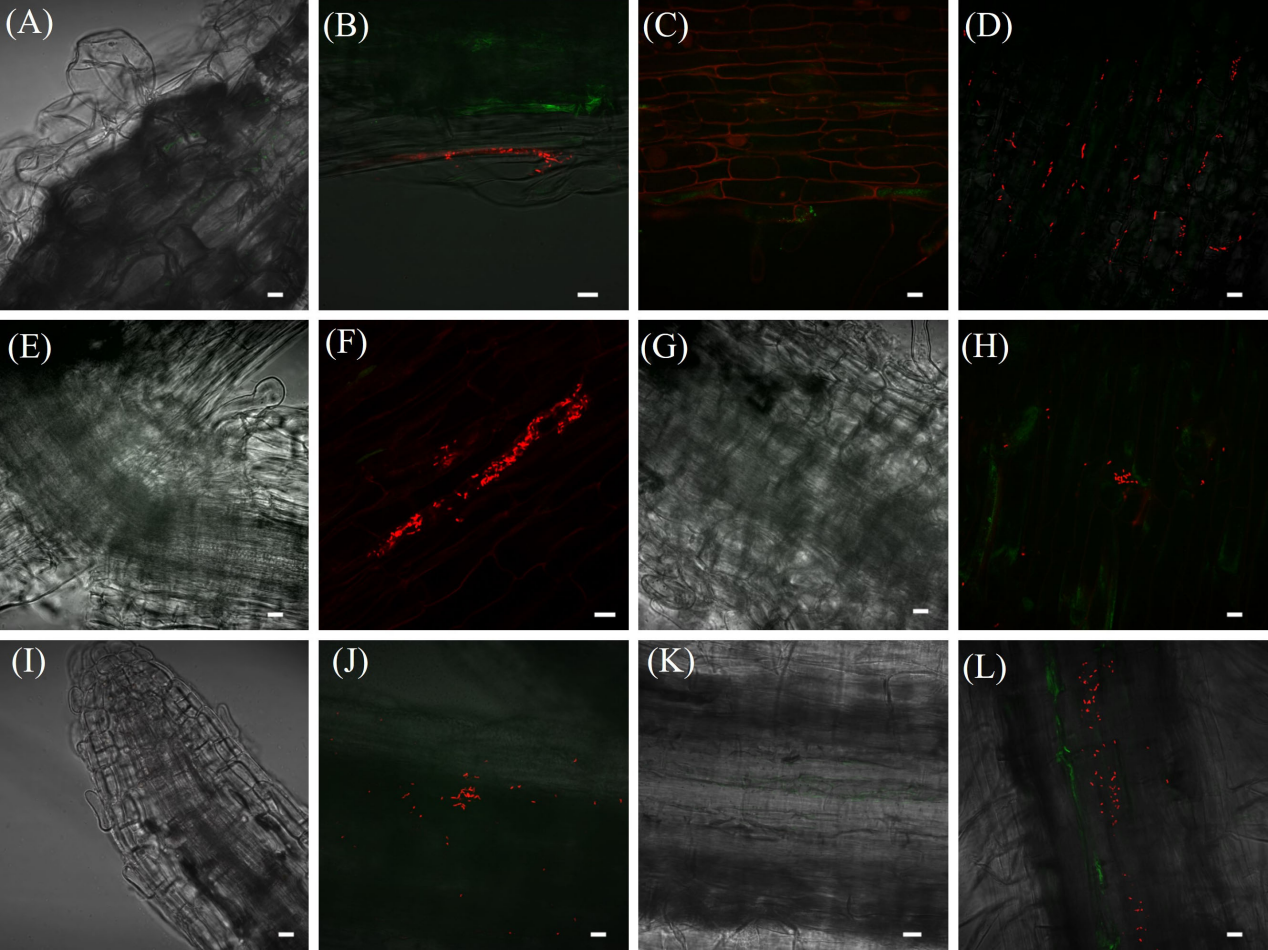
Microscopy images of NRB142 (mCherry) colonizing *Brachiaria* roots. Photos: uninoculated *B*. *ruziziensis* **(A, E, I)**; inoculated *B. ruziziensis* **(B, F, J)**; uninoculated *B. brizantha* cv. Paiaguás **(C, G, K)**; inoculated *B. brizantha* cv. Paiaguás **(D, H, L)**. At 3 [Line **(A–D)**]; 7 [Line **(E–H)**]; 14 [Line **(I–L)**] days after inoculation. Scale bars represent 10 μm.

Microscopy analyses further validated these findings, showing significant bacterial aggregations attached to *Brachiaria* roots. Red fluorescent NRB142 (mCherry) cells were observed colonizing roots of *B*. *brizantha* cv. Paiaguás and *B*. *ruziziensis* (**Figures 2B, D, F, H, J, L**). In contrast, no fluorescent bacteria were detected in non-inoculated control plants (**Figures 2A, C, E, G, I, K**). The bacterial counting (CFU) analysis corroborated these observations, as no bacterial colonies developed on plates containing DYGS culture medium inoculated with dilutions from macerated roots of control plants.

## 4 Discussion

The screening for ACC deaminase-producing bacteria revealed a lower number of strains exhibiting positive activity in isolates from rhizospheric soil and disinfected roots compared to those from non-disinfected roots. These findings align with the study by Timmusk et al. (2011) that showed abundance of bacteria producing ACC deaminase in the rhizosphere of *Hordeum* sp*ontaneum* plants and almost null in soil samples. Despite the relatively low percentage of isolates displaying ACC deaminase activity, the results presented here are consistent with other studies. For example, Jalili et al. (2009) using a similar methodology to characterize *Pseudomonads* species, found that 14% of isolates exhibited ACC deaminase activity - 16% in *Pseudomonas putida* and 12% in *Pseudomonas fluorescens*. Likewise, Duraivadivel et al. (2020) observed a low percentage (22.4%) of ACC deaminase-producing bacteria within the total bacterial community associated with *Eichhornia crassipes*. Gupta and Pandey (2019) reported that approximately 30% of bacterial isolates from the rhizospheric soil of garlic (*Allium sativum*) exhibited ACC deaminase activity. Similarly, Zhang et al. (2020) identified a small fraction (9%) of bacterial isolates with ACC deaminase activity in the rhizosphere soil of jujube trees.

All tested strains exhibited ACC deaminase activity greater than 20 nmol α-ketobutyrate mg^-1^ protein h^-1^, a threshold identified by Penrose and Glick (2003) as sufficient for a bacterium to growth in medium ACC-containing media and potential plant growth-promotion. The highest detected activities ranged between 80 and 100 μmol α-ketobutyrate mg^-1^ protein h^-1^. However, Penrose and Glick (2003) also noted that bacteria with elevated ACC deaminase activity (300 to 400 nmol α-ketobutyrate mg^-1^ protein h^-1^) do not necessarily stimulate greater root elongation than those bacteria with lower enzyme activity. The results presented here agreed with those found by Li et al. (2011), where *Pseudomonas* and *Herbaspirillum* species exhibited ACC deaminase activity comparable to the values observed here. Furthermore, ACC deaminase activity has been increasingly reported in the genus *Herbaspirillum* (Blaha et al., 2006; Rothballer et al., 2008; Islam et al., 2009; Onofre-Lemus et al., 2009; Sun et al., 2010). Similarly, Niu et al. (2018) identified ACC deaminase activity in *Pseudomonas* species isolated from millet, though the activity levels (39.40 µmol) were lower than those observed for *Pseudomonas cremoricolorata* (49.30 μmol α-ketobutyrate mg^-1^ protein h^-1^). According to Li et al. (2015), bacterial growth in an ACC-containing medium and the detection of low ACC deaminase activity do not guarantee the presence of ACC deaminase-producing bacteria. Therefore, unambiguous detection of the *acdS* gene is crucial for predicting enzyme activity and confirming ACC deaminase-producing bacteria. In the present study, the *acdS* gene was detected in nine bacterial strains. Chandra et al. (2018) employed primers (acdSf3 and acdSr3), as described by Li et al. (2015) to characterize bacterial strains isolated from soil samples collected in India. They successfully amplified a ~680 bp fragment specific to the *acd*S gene from the genomic DNA of *Pseudomonas* sp. DPB13, DPB15, and DPB16.

Polyethylene glycol is widely used in assays to simulate water stress in plants by lowering the water potential of the rooting medium, subsequently affecting plant water potential (Lawlor, 1970). In this study, PEG 8000 was utilized to assess the effect of ACC deaminase-producing bacterial inoculation on *Brachiaria ruziziensis* under *in vitro* water stress conditions. The results demonstrated that bacterial inoculation significantly enhanced root and shoot development in plants treated with strains NRB142 (*P*. *silvatlantica*), NRB223 (*A*. *melinis*), and BR11790 (*H*. *frisingense* GSF30^T^). Similarly, Kang et al. (2014) reported the successful colonization of cucumber plants by *Burkholderia cepacia* SE4, *Promicromonospora* sp. SE188 and *Acinetobacter calcoaceticus* SE370. These rhizobacteria conferred protection to plants grown under PEG-mediated stress, promoting increased fresh biomass accumulation in both shoots and roots biomass compared to non-inoculated control plants. These findings agreed with the results observed in the present study with *Brachiaria* genotypes, reinforcing the potential of ACC deaminase-producing bacteria in enhancing plant tolerance to water deficit stress.

Barnawal et al. (2017) reported that wheat plants inoculated with *Bacillus subtilis* strain LDR2 produced significantly higher biomass production compared to non-inoculated plants, both subjected to PEG 10000-mediated stress. Similarly, Govindasamy et al. (2020) observed that *Sorghum bicolor* plants inoculated with plant growth-promoting bacteria displayed a notable increase in shoot and root length under PEG 8000-mediated stress conditions.

An essential factor in mitigating of the water deficit stress is the plant colonization by ACC deaminase-producing bacteria. In this study, we demonstrated that strain NRB142 (*P*. *silvatlantica*) successfully colonized *Brachiaria* plants endophytically, with bacterial counts showing indicating substantial colonization the inoculated *Brachiaria* roots. This finding corroborated with those of García et al. (2019), who documented the colonization of barley plants (*Hordeum vulgare* L.) by *Paraburkholderia tropica* MTo-293 through colony counting and confocal microscopy. Similarly, Ramirez-Mata et al. (2018) employed the *mCherry* reporter gene to monitor *Azospirillum brasilense* colonization in wheat plants (*Triticum aestivum*), while Ferreira et al. (2020) evaluated *Rhizobium* sp. BR 10268 colonization in sugarcane mini-setts. These studies highlight the effectiveness of molecular and microscopy-based techniques in assessing bacterial colonization. In addition, it further supported the use of such methodologies to confirm the colonization of *Brachiaria* genotypes by the ACC deaminase-producing strain NRB142 (*P*. *silvatlantica*).

## 5 Conclusion

Plant growth-promoting bacteria with ACC deaminase activity plays an important role in enhancing plant tolerance to water deficit stress, thereby improving biomass production and yield. Our study showed that some bacterial strains exhibited notable ACC deaminase activity *in vitro* and demonstrated the ability to protect *Brachiaria* plants under PEG 8000-mediated stress conditions. The results indicated that ACC deaminase-producing strains improved physiological and agronomic parameters of *Brachiaria* plants, including shoot and root length as well as enhanced biomass accumulation under water stress conditions.

These findings suggest that diazotrophic bacterial strains containing ACC deaminase could serve as effective inoculants to alleviate the negative impacts of water deficit stress on *Brachiaria* genotypes. However, further validation through greenhouse and field experiments is necessary to confirm the mitigation effects of these bacteria across different *Brachiaria* genotypes exposed to varying levels of water stress.

## Data Availability

The original contributions presented in the study are included in the article/supplementary material. Further inquiries can be directed to the corresponding author/s.
